# Mental health service user organizations in times of crises: adaptions, challenges and opportunities experienced by local associations during COVID-19

**DOI:** 10.1080/17482631.2024.2380360

**Published:** 2024-07-15

**Authors:** Katarina Grim, Urban Markström

**Affiliations:** a Department of Social and Psychological Studies, Social work, Karlstad University, Karlstad, Sweden; b Department of Social Work, Umeå University, Umeå, Sweden

**Keywords:** Mental health, user associations, advocacy work, peer support, digital transition, crises, COVID 19 pandemic

## Abstract

**Purpose:**

This study examines how local user associations of mental health service-user organizations were affected by the pandemic, in order to provide guidance to user organizations and surrounding actors on future advancements. The pandemic is used as a case to explore organizational resilience and digitalization during crisis.

**Methods:**

Data from focus group interviews and individual interviews with representatives of ten local associations were analysed using qualitative content analysis. A theoretical framework combining governance theory and organizational theory was applied.

**Results:**

Typically, associations swiftly restructured activities to support members to meet the urgent needs that arose, not least in relation to the digital transition. Simultaneously, face-to-face interactions was valued and some members became isolated. Public sector actors often did not prioritize collaboration, and the associations had limited agency and influence in advocacy activities.

**Conclusions:**

User organizations can play an important role in times of crisis. Surrounding social systems should provide resources to counteract resource dependencies and allow organizations to develop operating reserves. They should value collaboration and establish collaborative practices to ensure a readiness to utilize the organizations’ capacities when needed. User organizations should have control over future developments, both to harness the potential of digital connectivity and to prevent a digital divide.

## Introduction

User involvement is crucial for sustainable support systems (Grim et al., 2022; Näslund et al., 2018), and maintaining opportunities for people to be included in participatory work is particularly important in times of crisis (Beresford et al., 2021). During the pandemic, reports showed that the social distancing measures required during the pandemic were associated with social disconnection and increased ill health among people with mental health problems (Brülhart et al., 2021; Moreno et al., 2020; World Health Organization, 2022). In contrast to many other countries, the Swedish government did not impose a total lockdown of society as a whole (Lovik et al., 2023). However, periods of strict restrictions, including avoiding public transport and crowded locations, and self-isolation for at-risk groups and people aged 70 and over, still affected the lives of Swedish citizens (Valeriani et al., 2020). Simultaneously, digital forms of connectedness and participation were developed, creating conditions that increased access to collective forums for some groups but reduced accessibility for others (Roeg & Weerman, 2020; Williams & Tembo, 2021).

Recent studies highlight the ambiguity of these conditions for user involvement under the constraints of COVID-19, describing how remote working has different effects on people and challenging the assumption that social distancing automatically means social isolation. While it increased opportunities for some groups to participate, such as those facing difficulties in travelling, others were excluded due to limited computer access or digital literacy, contributing to a digital divide (Beresford et al., 2021; Roeg & Weerman, 2020).

Accordingly, while the newer, distanced ways of working have the potential to enhance user involvement, there are issues that need to be addressed to enable broadened participation and to counter the risks of digital exclusion for certain groups.

### The mental health service user movement

In the field of mental health, the organized user movement is a key stakeholder in promoting user influence and in developing services and systems that are democratic, recovery oriented and efficient (Näslund et al., 2020). Non-profit organizations have multiple purposes, and their role is generally characterized by the goals of member support, knowledge dissemination and political advocacy. Member support typically includes social activities, mutual aid activities and service commission. They tend to be of an open support and general counselling nature, i.e., mainly informal, non-bureaucratic or authority-based services such as café evenings, excursions, peer support groups and helplines (Näslund et al., 2020). Moreover, courses focusing on coping strategies are typically provided, often led by volunteers with experience of mental illness or of being a relative. In some cases, service interventions are delivered in collaboration with public organizations and professionals, such as counsellor-led discussion groups (Näslund et al., 2018). Political advocacy involves pursuing issues of interests of their members and seek to influence public opinion, policy and social development as well as making demands on politicians and officials. Activities for knowledge dissemination may target various audiences, such as the general public, members, professionals and politicians, and thus intersect with the goals of both member support and political advocacy (Markström & Karlsson, 2013; Näslund et al., 2020).

The welfare system in Sweden is characterized by a large public sector responsible for providing services to citizens, to which the voluntary sector plays a complementary role. The Swedish mental health service user movement consists of a multiplicity of non-profit organizations where the financial and administrative control resides with members that are predominantly service users or their relatives. These organizations, with their local associations throughout the country, rely on unpaid efforts and are partly supported by government funding (Näslund et al., 2020). They vary in terms of organizational characteristics, political orientation and target groups (Markström & Karlsson, 2013; Näslund et al., 2020). While some unite around the identity of having lived experience of mental ill-health, others unite around more distinct identifications, such as specific diagnosis groups (Näslund et al., 2018). The seventeen of the largest organizations belong to the umbrella organization The National Partnership for Mental Health (NSPH). Typically, local organizations are important actors in advocacy, negotiation and cooperation with authorities such as social services and mental health services. In most cases, these processes are put into practice in different types of local cooperation groups that are established by the authorities and include different stakeholders (Markström & Karlsson, 2013).

In the aftermath of the pandemic, the associations need to maintain and develop the support for their members in a communication landscape that is characterized by a high degree of digital connectivity. There is also the issue of ensuring that the continued development of mental health services includes the target groups of the services in development processes. A current challenge is to strengthen and sustain participation, by incorporating both traditional and emerging approaches to involvement (Beresford et al., 2021).

The current research project focuses on activities in *local* associations. These include service development activities, user councils, peer support groups or information campaigns. All of these approaches serve to strengthen readiness for increased co-production and political advocacy. Accordingly, the current study has the ambition to increase knowledge about political advocacy and member support in times of crisis, which can further support the development of user connectivity and involvement in a post-COVID world.

The overall aim is to examine how the work of local user associations has been affected by the pandemic. By exploring the adaptions that have been made, challenges and opportunities can be identified that may inform strengthened user involvement in the times that follow after the pandemic. Research questions posed are:

- How have member activities been affected?
- How has advocacy work been affected in relation to the care and support system?
- What strategies and methods have been applied in response to the pandemic?
- What was learned from these experiences in relation to future advocacy work?

### Theoretical framework

The local perspective of this study allows for a distinct actors’ perspective. In line with organizational and new institutional theory, Arvidson et al. (2018) and Johansson et al. (2019) suggest that non-profit actors are neither passive recipients of political decisions nor bound by institutional structures, but can be understood as active strategic actors. This allows for a scrutiny of the considerations, negotiations and contradictions that may arise in the associations’ daily activities, in terms of how they work with their representation towards municipalities and officials, what strategies they use to exercise advocacy, and also what challenges they face in securing resources and upholding their core values.

Accordingly, in order to shed light on aspects of the associations’ circumstances, strategies and agency within the broader societal context, we apply concepts from a theoretical framework specifically developed by Arvidson et al. (2018) and Johansson et al. (2019) for the analysis of non-profit organizations based on the keywords governance and adaptation. The framework combines governance theory and organizational theory providing a tool for examining the interactions between institutional environment and organizational agency, as well as the complexities inherent in the relationship between the non-profit and the public sector.

The framework provides three building blocks for analysis:

1. The institutional environment, which encompasses circumstances in the external setting, which in the case of non-profit organizations is dominated by the public sector. It includes both structural and cultural factors linked to established societal decision-making pathways, norms and role expectations.
2. Characteristics of relations, referring to the interdependence that occurs with external actors where the qualities of affiliation set the conditions for the exchange of resources in the form of funding, knowledge, services and accessibility.
3. Intra-organizational factors, which include internal structures (capacity and connectivity) and ideological orientation (conflict- or consensus).

## Methods and materials

### Design

A collective case study was conducted involving 10 local Swedish user associations, all members of NSPH. The associations were purposively selected in order to capture a variety of action repertoires and experiences of user involvement during the pandemic. Data were collected through focus group interviews (FGIs) and individual interviews.

### Recruitment and participants

Five organizations participated; RSMH (National Association for Social and Mental Health, in Swedish: Riksförbundet för Social och Mental Hälsa), the Schizophrenia Association, SHEDO (Self-Harm and Eating Disorders Organization), Attention and Balans. See Table 1 for the target groups of each organization. For each of these five, two local associations were selected in different parts of Sweden, taking into account the geographical spread, as well as the size and demographics of the municipalities.

**Table 1.** Overview over organizations, local associations, settings and participants of the FGI:s along with specification-codes for quotes.

Mental health service user organization	Focus	Settings, participants (*N* = 46) and codes for quotes	
		Board members (*N* = 25)	Members (*N* = 21)
Attention	Neuropsychiatric disabilities	*Association 1.*	
		Performed at a library (*n* = 2) (Of which one was chair) A 1a.	Digitally performed (*n* = 2) A 1b.
		*Association 2*. Both digitally performed	
		(*n* = 3) A 2a.	(*n* = 1) A 2b.
Self-Harm and Eating Disorders Organization (SHEDO)	Self-harm and Eating disorders	Both digitally performed	
		(*n* = 2) (Of which one was chair) B 1a.	(*n* = 2) B 1b.
Balans	Affective disorders	*Association 1.*	
		Digitally performed (*n* = 4) (Of which one was chair) C 1a.	Performed at the premises of the association (*n* = 2) C 1b.
		*Association 2.*	
		FGI performed in mixed group on the premises of the association (*n* = 5) (2 board members of which one was chair, and 3 members) C 2.	
The Schizophrenia Association	Psychosis disorders	*Association 1.*	
		Both performed at the premises of the association	
		(*n* = 2) (Of which one was chair) D 1 a.	(*n* = 4) D 1 b.
		*Association 2.*	
		Both performed at the premises of the association	
		(*n* = 3) Of which one was chair) D 2 a.	(*n* = 4) D 2 b.
National Association for Social and Mental Health (RSMH) Riksförbundet för Social och Mental hälsa	Does not focus on any specific diagnosis	One FGI performed in mixed group (*n* = 8) (5 board members and 3 members) at the premises of the association E 1.	
		E 2. Individual interviews with: A chairperson performed digitally (*n* = 1) E 2. A board member—on the premises of the association E 3. (*n* = 1)	

Through initial contacts with the chairpersons of the boards of the national organizations, we identified chairpersons of local associations that might be suitable for inclusion in the study. We aimed to strategically select local associations that we believed might have thrived during the pandemic as they already conducted much of their communication and activities online, those that might have suffered, as they were previously doing all their activities in face-to-face meetings, and those in between.

These chairpersons were then contacted and asked to help recruit two strategic samples of participants for separate FGIs a) board members who were actively involved in arranging membership activities as well as in collaborative work with municipalities and regions, b) members of the local associations. All the chairpersons initially contacted expressed interest in participating and facilitated the recruitment process. They shared information about the study and the opportunity to participate through their regular information channels, such as letters, emails and social media, which reached all members. They facilitated contacts between the researchers and potential participants for the FGIs, as well as handling the practical aspects of locations and invitations. Participants could choose between face-to-face or virtual meetings for the FGIs. In one case, despite efforts, it was too difficult to recruit and arrange FGIs. To compensate, individual interviews were conducted with one chairperson and one board member from the same association.

A total of 46 interviewees participated in the FGIs, including 27 women and 19 men. Table I provides an overview of the associations, their membership groups, and the settings and number of participants in each focus group. The sample of participating members generally reflected the characteristics of the general membership of each association. All chairpersons who had facilitated the organization of the FGIs participated.

### Data collection

Of the associations that opted for physical meetings, some had their own premises, while others had to find other suitable venues. Typically, these FGIs took place in conjunction with board meetings, member meetings or “café evenings”. Although the aim was to have separate FGIs with board members and members, allowing participants to freely express their expectations and feelings about the other group, some were conducted with mixed groups. In some cases, participants felt that their roles overlapped, and in others, some explicitly wanted to participate together.

Pre-developed interview guides included open-ended questions aimed at exploring interviewees’ views and experiences on the key issues of the study. Questions were themed around the three main functions of non-profit organizations: member support, knowledge dissemination and advocacy.

The interviews were conducted in the spring of 2022. The second author conducted interviews with the associations identified in Table I. by A and B, and the first author conducted interviews with the associations identified by C—E. The audio files were shared between the authors immediately after the interviews for ongoing monitoring to promote consistency in the interviews. Interviews lasted between 45 and 90 minutes and were audio-recorded and transcribed verbatim. Informed consent was obtained verbally from participants in digital interviews and in writing from participants in face-to-face interviews. *Ethical approval* for the study was obtained from the Swedish Ethics Review Board (2021–05667–01).

### Data analysis

Data were analysed using qualitative content analysis (Graneheim et al., 2017). An abductive approach was applied, incorporating both inductive and deductive methodology at different stages of analysis (Graneheim et al., 2017). The analysis process began by repeated review of the data to gain a sense of the whole. Next, data was read word by word to derive codes by marking words, sentences and phrases that seemed to capture key thoughts or concepts. Most of the codes related to various activities and functions of the associations and captured a variety of contextual circumstances and how they were affected by social distancing and the digital transition. Some codes also captured reflections on future developments.

In the course of the analysis, it became evident that a predominantly chronological sorting of the main categories was the most natural and would provide the most clarifying organization. The first main category includes comments on the *changed conditions and actions taken*, This is followed by the main categories *recourses and resource shortages, positive consequences, negative consequences and looking ahead.*

In a next step, the codes belonging to the each of the main categories were sorted into sub-categories, three of which were based on the main functions typically associated with non-profit organizations, (as thermalized in the interview guide), namely of providing *member support*, conducting *knowledge dissemination* and of engaging in *advocacy work* to influence public policy and officials. As a significant propotion of the circumstances and activities concerned *administrative and operational work*, this was chosen to form a fourth sub-category.

Each of these four sub-categories is described under each of the six main categories outlined above, providing a predominantly descriptive account of the interviewees’ experiences and reflections. We then apply concepts from Johansson et al. (2019) and Arvidson et al. (2018) theoretical frameworks, as outlined above, to further analysis how the pandemic has affected activities in the associations from an actor’s perspective. The written results section was reviewed through the lens of the included constructs and was roughly coded by highlighting excerpts that related to each of them. Passages referring to the roles and functions of organizations in relation to other institutions were connected to *institutional environment*, passages referring to interdependencies and various actors’ commitment and conditions for collaboration were connected to *characteristics of relations*, and passages referring to the resources, infrastructure and ideological orientation of organizations and associations were connected to *intra-organizational factors.*

In order to indicate the source of the FGI quotations, they are marked with the codes given in Table I. (The capital letter (A—E) indicates the organization. The number that follows (1–2) indicates the local association. The lowercase letter at the end indicates whether the quote comes from a board member (a) or member (b).

## Results

### Changed conditions and actions taken

The conditions for the digital transition varied across organizations. As regarded activities for *member support*, those that already conducted much of their activities digitally, made the transition swiftly. SHEDO, which initially started as an online peer support movement, was active on social media platforms that attracted many young people with mental health problems. During the pandemic, they saw an increase in website traffic and demand for their support chat. Similarly, the other associations experienced an increased need of counselling and social activities. In accordance, representatives from Attention highlighted how the pandemic heightened anxiety among individuals with neuropsychiatric conditions, with the fear of infection intensified by their tendency to think in “black and white” terms, and how anxiety was “exacerbated by loneliness and not having a counterpart with whom to vent their worries and discuss relevant protective measures” (A1 a). Similarly, a representative of a schizophrenia association described how their members who typically suffered from marginalization and unemployment, were now left in total isolation, noting “how delusions and hearing voices were often intensified by being alone” (D2 a).

Many interviewees emphasized how the isolation experienced by members was compounded by the reduction or closure of social support services provided by the public health and social care system, obstructing social interactions and activities that were crucial for these individuals. These concerns are reflected in the following quotes, in which the interviewees describe the lack of understanding of the risk of loneliness on the part of public actors and the slow implementation of secure digital solutions:

For years, two caregivers would come at the same time, and suddenly there was only one. And you reduce it for the people who you thought for some reason did not need that contact. Then it was removed completely. So there were quite a lot of complaints about it, because it removed in a time when it was really needed (E 2).

The failure to solve technical problems in organizing opportunities for social interaction demonstrates a lack of understanding of the importance of counteracting isolation:

There were no educational and supportive courses for patients and families, because it was never started, because it took quite a long time for Stockholm mental health services to be able to handle confidentiality with Teams (C 2a).

In describing how they had adapted their member support in response to the situation, SHEDO representatives reported that they had cancelled some planned digital lectures for staff groups in favour of organizing lectures for patients and their families. Associations with limited experience of online activities described how they were unprepared and how many group activities were cancelled for up to a year. To support their members, some actively called, texted or wrote to their members, and others expanded their telephone support capacity. One association purchased second-hand computers to provide to members who did not have access to one, along with basic IT training and support.

In some cases, even though members belonged to groups at risk of serious illness or death, the risk of isolation was judged to outweigh the risk of infection, and with precautions such as physical distance, hand sanitizers, and limited numbers of participants, some peer support meetings continued without interruption. In particular, when talking about weekly support groups or café evenings, interviewees from several associations spoke in terms of them being “sacred” (E1), “going on even if it’s Christmas Eve” (E2) “a lifeline” (D2 a) or “life-sustaining” (C2 a). In the main, however, all the associations adhered closely to the guidelines of social distancing. Weather permitting, many organized outdoor social activities such as walks, picnics, and barbecues. Some of the peer support groups were also held outdoors.

Even the primarily online SHEDO association typically conducted counselling training in face-to-face meetings. In the face of the pandemic, they were reluctant to conduct them digitally because face-to-face meetings provided a safe environment in which participants could share personal experiences and role-play sensitive issues, and facilitators could pick up on any discomfort or anxiety and provide support. However, given the choice of conducting them digitally or not at all, they were conducted digitally, which interviewees said worked well.

Members describing the transition to digital formats noted that a variety of helpful guidelines were in place to ensure a safe space for discussion:

It works great. Well, we did that now when we had these support groups, that you, you can press, raise your hand, on this button. And then, as we said at the time, everybody gets to talk to the point. And you don’t interrupt and you don’t give advice unless they ask for it (C 1a).

As for the activities involved with *knowledge dissemination*, many reported that scheduled lectures had been redesigned as online events. Representatives of SHEDO noted that their planned cross-country lecture tour, which was originally intended to be a ticketed event, was instead offered as an open online event. Representatives of several associations reported that they had arranged for lectures scheduled to be held at their local facilities to be broadcast nationally instead, making them accessible to members of all their local associations nationwide. However, some noted that not all lecturers were comfortable giving digital lectures, but wanted an audience with whom they could interact.

Regarding the impact of the pandemic on *advocacy work*, board members commonly described how user councils were affected. Many user councils were cancelled for a period of time before moving to digital meeting formats. Instead, some board members reported how they engaged in other forms of public pressure, such as writing opinion pieces for campaigns and newspapers.

As regarded the *administrative and operational work*, board meetings and federal councils typically moved to digital platforms. In one of the schizophrenia associations, where board members tended to be older, board meetings were held by telephone. Attention representatives reported how the uncertain situation with constant new offers regarding restrictions meant that they had to plan on a short-term basis, which required them to hold more frequent board meetings. “There had to be a plan A, B, and C all the time” (A1 a). For most associations, annual meetings were typically postponed, held digitally, or in a hybrid meeting format.

### Recourses and resource shortages

Some board members noted that the digital transition had reduced travel and accommodation costs associated with activities for *member support* and *knowledge dissemination*. However, associations often had to purchase computers, cameras, and screens, as well as licences for digital platforms, which was a financial burden.

When discussing *administrative and operational* work, board members described how the pandemic put increased pressure on their associations, which typically had limited resources to cope. The demands of social distancing required increased efforts to support members. Representatives from RSMH and Balans described how some board members worked hard to provide extra telephone support, and a representative from SHEDO noted how they had to recruit more volunteers to extend opening hours and staff the chat room.

Some described the additional efforts involved in organizing outdoor gatherings with safe catering of food and drink, and setting up and navigating digital platforms. Most of the participating associations operated entirely on an unpaid basis. Many noted that this voluntary work was particularly demanding in times of crisis. A representative of Attention noted that the pandemic also created a strained family situation for many board members, who typically had children or other family members who suffered from increased illness during this time. However, among the larger associations, particularly those based in metropolitan areas, some had hired staff and established partnerships with surrounding care- and support actors, allowing for more extensive support interventions such as vocational training.

It was clear from the reports of the participating board members, that managing the digital transition was person-dependent. They often referred to individual board members who were tech-savvy and had a special interest in setting up equipment for virtual meetings as crucial for the association to be able to offer online activities. In addition, some described it as time-consuming and difficult to help members connect to and navigate the online tools.

### Positive consequences

In line with recent research (Beresford et al., 2021), it was clear that working online had had some positive effects and provided opportunities for wider access and inclusion. Many noted that those living outside of cities had better opportunities to participate.

As regarded activities for *member support*, certain course programmes that were normally offered locally were now being offered through digital platforms, allowing members and individuals from neighbouring regions or even those in regions without a local association to access these events.

In addition, it was observed that virtual meetings provided a more comfortable environment for individuals who, regardless of the pandemic, had fears related to travelling by subway or engaging in social encounters,

Those who didn’t feel like getting on the subway for fear of COVID could participate. Maybe in their normal state they wouldn’t have dared to take the subway because they didn’t like going out into the city, you know, meeting people and all that. So we lost a certain group of people, but we gained another group of people who appreciated these Zoom meetings (C2 a).

Similarly, people suffering from depression found it easier to participate. As one member noted, “you could join in half lying on the bed” (C2b). Some board members noted how the situation had compelled people to learn how to use new technology and remarked that they had been pleasantly surprised by their members’ ability to learn and adapt.

Among members, it was clear that the digital transition affected people differently, in part depending on their primary purpose for being a member. Those who were primarily focused on increasing their knowledge of mental illness, learning coping strategies, and listening to the recovery journeys of others with mental ill-health, generally preferred the easily accessible digital format. One member who expressed that she needed information rather than social companionship appreciated that a support group that transitioned to a digital format had included members from neighbouring associations, providing diverse input on coping and recovery strategies.

It’s not really the companionship I need, it’s the information…knowledge about this and the experiences of others (C 1b).

Indeed, it was noted that most activities were focused on *knowledge dissemination*, whether aimed at people living with the illness, their families, or professionals, benefited from the digital transition in that it allowed for greater participation by removing geographical and space constraints. Some noted how lectures could be arranged in collaboration with their county or national organizations, drawing on the technical resources and expertise of those more advanced in this regard. A member of one association noted that she appreciated that some digital lectures were accessible online and could be viewed multiple times.

While some associations experienced a decline in membership during the pandemic, others reported stable or even slightly increased membership. A board member of one of the schizophrenia associations reflected that this crisis, which led to more online activities, may have increased the reach of information and “boosted a declining interest in non-profit organizations” (D2 a).

With regard to *advocacy work*, many board members expressed how they appreciated not having to travel to attend user councils and how user councils were more effective and accessible to a wider range of people.

Board members also expressed the hope that the wider community will have noticed how the associations have served an important purpose in times of crisis, when demand for support increased and public care and support services, already under strain before the pandemic, faced major challenges in maintaining their capacity. One board member noted that the pandemic had raised a general awareness “that civil society is doing good and important things” (B1 a) and hoped that this would increase their chances of receiving more resources.

Another hope expressed by interviewees was that professionals, who they noted typically had low expectations of the competence of mental health service users, would recognize that the associations had been able to adapt and use the digital tools, thereby contributing to a change in attitudes. As one board member noted,

I don’t think anyone now doubts that just because you have a mental illness you can’t use Zoom or something like that. So I think it has probably become a learning process for the professionals as well. A lot of us in associations became very comfortable with platforms like Zoom and Teams because of our board meetings and annual meetings… (B1 a).

As for *administrative and operational work*, one SHEDO representative reported that mentor and counsellor training, which had previously been held in metropolitan areas, was now available nationwide. Another noted how they had received inquiries from individuals who had attended open lectures and were interested in becoming local representatives in sparsely populated counties. Board members generally observed an increase in attendance at formal meetings, such as board meetings, as it became possible to attend even if one had a cold or was travelling.

In addition, board members from several associations expressed pride and a sense of empowerment that they, as an association, had successfully navigated the crisis and effectively managed the digital transition. In the quote below, a board member of a SHEDO association expresses how this accomplishment had strengthened their sense of community:

I think it strengthened us, the fact that we never had to cancel, but we were able to adapt. It strengthened the community to know that we did this together. And it felt great that way (B 1a).

### Negative consequences

Representatives from all of the associations, which typically provided *member support* in physical meetings, expressed a number of concerns related to social distancing and the digital transition. Many activities performed under ordinary circumstances, such as discussion groups and study circles, were noted to provide forms of support that were not accessible through the public health care and support system, and thus provided an important complementary service.

Moreover, it was noted how the association provided interventions that the public system failed to deliver despite being within its area of responsibility, such as next-of-kin support. As described in the following quote:

It is very much in demand, our counsellor-led discussion group, and that is partly because there is a gap. It’s really a regional responsibility to make sure that you get support as a relative, but it doesn’t really work that way. It’s true that there should be relatives’ counsellors … well-trained counsellors in the municipality, but they don’t exist, so we go in and do it (D2 a).

According to board members, some members were reluctant to participate in outdoor activities or use public transportation for fear of infection. Many noted that members who were unemployed or living alone and who valued the social aspects of group activities suffered from social distancing. For many, the face-to-face meetings provided motivation to dress and leave the house. In the following quote, a board member of a Balans association expresses concern for members, some of whom, prior to the pandemic, had only summoned up the courage to join the association’s community, which provided access to human closeness and undemanding interaction:

… yes, who sat there and maybe weren’t very active, but at least dared to come here. And they were left alone, now, at home. And it broke my heart that they couldn’t do anything … those who had been ill for a long time, who had finally come to a community where you could just be. It must be a tragedy for that kind of person. …those who are a little closed off and maybe don’t socialize as much (C2 a).

Although virtual meetings were more accessible for many, interviewees typically expressed that they could not replace the trust and relationship-building elements of physical meetings. In the following quote, a board member metaphorically describes the qualities lost:

Things become flat…two-dimensional. You don’t feel that depth and warmth and spaciousness. …the feeling of a personal encounter…the community. You don’t get to know each other in digital meetings (C2 a).

Some members who noted that they felt uncomfortable with cameras and did not want to use zoom, for example because they did not know who might be listening in the background if they were being recorded, expressed how they felt excluded and sometimes ostracized for not “keeping up with the progress” (E 1). As one member put it:

I don’t trust the world of technology…I’m scared…I don’t want to, I don’t want to! And even though this is an association for people with mental illness, there is no understanding or compassion for such feelings (E1).

It was commonly observed that the pandemic served to exacerbate the digital divide for many members. Typically, the elderly and those without computers were observed to suffer from exclusion. Poverty was noted as a contributing factor.

A board member observed how people over 70, who were advised to strictly limit their social contacts and avoid places where many people gather, suffered intensely from loneliness:

Elderly people felt completely outside the world because no visitors were allowed. No one could come. If you had children, they couldn’t come. No one called or kept in touch (E2).

Among the board members, it was generally noted how new members were usually recruited through open cafes or by representatives attending various events in the external context. When the pandemic prohibited such activities, recruitment was hampered.

Although it was observed that the digital transition supported many aspects of *knowledge dissemination*, it was commonly noted that many social and interactive qualities were lost. Some described how local lectures often included time to mingle and discuss the topic of the lecture over coffee and snacks. One member noted how she missed the “full experience” and “ceremony of going to a lecture” (D1 b). In addition, some expressed that they felt less comfortable asking questions in a digital environment.

Many of the board members were also aware of the loss of social aspects of *advocacy work*. They expressed how they missed the opportunities for small talk that are present in physical meetings, a factor they identified as important for building relationships, which in turn was identified as an essential factor for gaining influence in planning and decision making.

Furthermore, board members often described how public organizations, authorities, and services had cancelled all user councils and noted that these organizations did not appear eager to find alternative ways to communicate. They described how they had reached out and requested user council activities, but had not received a response. Some even suspected that the pandemic was being used as an excuse to avoid user councils. These sentiments are reflected in the following quotes:

That it had to, it had to stop during the pandemic and that it wasn’t the case that the regions were so desperate, oh, now it’s a pandemic, now we have to find another way for you to work with us. But it got pretty quiet (C1 a).

Board members of the Balans associations described how they continued to pursue their advocacy interests by informing members of the obligations of public care and support services to arrange and provide coordinated care plans, crisis plans, and training for patients/users and their families. One board member described how, by “agitating” members to demand” (C 2a) these interventions, they created pressure on services to meet their responsibilities.

Some associations that typically engage in user-focused monitoring (UFM), a method of evaluating mental health services conducted by people with lived experience of mental illness, found that this form of user advocacy was impossible during the pandemic as it required physical meetings with service users.

### Looking ahead

When considering the future, the majority of comments focused on *member support* activities. Board members typically noted the importance of reaching out to members and understanding their wishes and needs as regarded formats for meetings. In general, interviewees reflected that digital formats would be used to a greater extent than before the pandemic. In some associations, it was already clear that the majority of members wanted to continue with the more convenient and accessible digital meeting formats and that they had no choice but to follow their preferences, while in others, members yearned for face-to-face meetings. Some board members expressed that in certain instances they would encourage a return to physical meetings to ensure a safe and fruitful communication environment. In general, interviewees considered a variety of hybrid solutions as the way forward. When discussing the possibility of organizing support groups or study circles digitally and physically, with alternating forms of meeting, it was typically noted that it is important to hold the first few meetings in the face-to-face form in order to support the opportunity for participants to build trusting relationships and a safe community for sharing sensitive issues.

The pandemic years demonstrated the important role of associations in supporting people in times of crisis. Several had made great efforts to provide telephone support to lonely and distressed members. Some hoped to be able to set up not-for-profit helplines, in part to be prepared for future crises, but also to be able to work preventively. The organization of SHEDO, which offered a mentoring programme involving one-on-one contact with mentally ill people or their families, was in the process of determining whether video, telephone, chat, or email would be the best form of communication. Some associations planned to create digital platforms on Facebook for members to meet and share experiences.

While the majority of participating board members described how the digital transition had expanded the associations’ repertoire of activities, some expressed concern that they had lost touch with the many members who were unable or unwilling to participate in digital activities and were unsure whether they would return when physical meetings resumed. As one board member noted:

…members we haven’t seen in years. We don’t know what’s happened to them. Those who used to come regularly have disappeared. We don’t know if they’re coming back…or how they’re feeling and how they’ve been doing (A1 a).

With regard to *knowledge dissemination* activities such as conferences and lectures, interviewees typically reflected that some will be held online and some onsite. Digital lectures do not strain the associations’ budgets for travel and accommodation costs and are accessible to a wider audience. Board members typically discussed the possibility of hosting hybrid events, offering a choice between attending in person or virtually. However, it was noted that it is often difficult to facilitate interactive activities with both in-person and online participants, and some suggested that online participants should only be able to listen.

When discussing future *advocacy work*, board members hoped that user council meetings would alternate between digital and physical formats. They described an imbalance of time and effort often existing between themselves and the professionals, as they had to take time off work and travel while the professionals attended during their paid working hours on their own premises. As digital meetings do not require much time away from work, many noted that they would be able to attend more of these meetings if held in digital formats. On the other hand, they emphasized the importance of physical meetings for building relationships and trust.

In addition, board members described how the effort and resistance they faced in gaining influence in user councils, both in ordinary and pandemic times, led them to prioritize member-focused activities and other ways of exerting influence. As one board member reflected,

We have to prioritize, and we have prioritized member activities over advocacy because we feel it is so difficult to have influence. It is extremely difficult. And that we affect more people’s lives by having good activities and good support for members than by trying to have a meeting with managers (A 1a).

As for *administrative and operational work*, most associations would continue to hold board meetings online. However, some of the board members who had retired from the work expressed a strong desire for a return to physical meetings.

## Discussion

In line with the aim of the study, the analysis of the accounts of the members and board members of the local associations shed light on the conditions and actions of the associations during the COVID-19 pandemic and the impact of the crisis on their ability to fulfil their roles as disseminators of knowledge, advocates of their interests and supporters of their members.

By examining events and activities during the restrictions, the analysis has highlighted a number of problematic issues and facilitating factors in the associations’ relationships with their external environments and actors, as well as in their internal structures and core purposes. Accordingly, the application of constructs from the theoretical framework built from the three constructs of a) *institutional environment*, b) *characteristics of relations*, and c) *intra-organizational factors* (Arvidson et al., 2018; Johansson et al., 2019) may shed further light on priorities that require attention and strategies that need to be kept in mind for future approaches.

When considering the conditions and actions of the associations in the context of their *institutional environment*, the findings align with previous literature (Karlsson & Markström, 2012), which describes a complementary role of non-profit mental health organizations in providing a variety of services that add to existing public services, e.g., in the form of peer support and knowledge dissemination. In international comparisons, previous studies have concluded that Swedish civil society organizations have traditionally been less service-oriented than those in, for example, the USA, the UK and Germany (Lundström & Svedberg, 2003; Trägårdh, 2010). However, recent literature highlights a shift within the Swedish institutional environment, whereby these organizations are increasingly expected to contribute as service providers (Arvidson et al., 2018). The current analysis draws attention to the strained conditions for public mental health systems even prior to COVID 19. In the face of the pandemic, the analysis highlights how the pressure on voluntary organizations intensified as access to public services was reduced. In harmony with research performed in the UK (Gillard et al., 2021), the current findings highlight how many public services became overloaded and some were cancelled or closed. This occurred the same time as their members’ need for care and support increased, underscoring the important role of civil society actors in times of crisis.

As for the *characteristics of relations* with external actors, various aspects of associations’ resource dependency were salient. Associations that were typically dependent on public funding from municipalities and regions were often the least vocal party in collaborative activities. In terms of service provision, activities in which they were usually involved, such as patient and family education, were cancelled despite strong requests from the associations. Associations were generally more eager to cooperate than public actors, and user councils were often down-prioritized by public organizations and authorities during the COVID-19 pandemic. As a result, when the pandemic struck, the associations had very limited capacity to advocate and influence, and had to adapt to the calls of the professional actors. However, the online user councils that *were* held made it easier for board members to participate, as they did not have to travel far in their free time, thus redressing one aspect of the typically unequal conditions of participation.

Issues related to *intra-organizational factors* represented the most salient aspects of the data. In general, associations struggled with a lack of resources in terms of money, time, and people. A lack of formal infrastructure and fixed, professionalized roles made the ability to manage digital transitions highly dependent on individual, tech-savvy enthusiasts. Despite these challenges, associations had typically strengthened their connectivity and expanded their repertoire of activities in terms of member support, knowledge dissemination, and advocacy. It should be noted that Sweden is highly developed in terms of access to the internet, computers and smartphones (Valeriani et al., 2020). This underlines how non-profit organizations in countries with lower socio-economic preconditions faced more demanding adaption challenges (Santos & Laureano, 2022).

With regard to their ideological orientation, the associations had a consensus perspective in relation to the formal care and support system and aimed to collaborate with public actors. In line with previous literature in the Swedish context (Karlsson & Markström, 2012), the associations acknowledged the knowledge perspectives of professionals as well as a medical model of mental health problems, but added a perspective beyond that. Their advocacy work rarely involved radical or confrontational approaches, but rather put pressure on public care and support actors to adhere to their own regulations and responsibilities, even in times of great pressure such as the pandemic.

## Conclusions: what needs to be done

We suggest that the insights provided by the current study may support the user movement to have choice and control over conditions in a post-COVID world. By exploring the adaptations that were made, challenges and opportunities can be identified that may inform strengthened civil society and user involvement in future developments. It is unclear whether the resilience shown by Swedish organizations and their associations was made possible by the fact that Sweden did not implement the strict lockdowns of many other countries, and thus did not have to redirect all communication to digital forums. Indeed, international studies demonstrate how the pandemic had devastating impacts on non-profit organizations (Akingbola, 2020) and how those that rely on in-person gatherings to deliver their programmes were the ones most severely affected (Kim & Mason, 2020). Concurrently, studies indicate that organizations in countries with the typically more stringent restrictions demonstrated flexibility, creativity and adaptability (Lebenbaum et al., 2024), in line with our findings.

The current findings indicate a number of issues that need to be addressed by actors within the service user movement as well as by welfare actors in the surrounding society. With regard to service user movement actors, it is evident that attention should be directed towards members and potential members for whom face-to-face meetings meet essential needs for social connectedness. The analysis shows how the associations typically took note of the new members who joined as a result of the increased accessibility provided by the digital transition, but how they tended to lose sight of the lost members, those who did not stay in touch, those who could not or did not want to participate in virtual meetings. In line with recent findings on the responses on a wide array of Swedish civil society organizations working with people in need of food, security and housing, which notes how face-to-face interactions are highly valued by those living precarious lives (Lalander & Herz, 2023), the current findings underscore the risk of exacerbating marginalization of already vulnerable groups. Not least, a too strong shift towards digital forms of meetings could exacerbate an already existing lack of diversity and equality in advocacy work (Ocloo, 2021), which is central to upholding the democratic aim.

In light of this, while user organizations continue to embrace the opportunities of the digital transition, they must remain vigilant to a possibility of a new normal emerging where digital is the default, and actively resist a normalization of social disconnection. These findings resonate with recent research reporting on experiences of remote support provision during the COVID-19 pandemic, not least in the voluntary mental health services sector, highlighting the importance of tailoring activities to a range of individual preferences, needs and resources (Rocha & Almeida, 2021; Worsley et al., 2023)

The study indicates that associations were typically able to restructure their member support activities to meet urgent needs arising from the consequences of the pandemic. Consistent with the findings of a previous study of Swedish patient organizations during the pandemic (Hjorth et al., 2021), the current analysis demonstrates how associations were in many cases able to make transitions more quickly than public actors. These findings corroborate the conclusions of a series of studies on Swedish civil society initiatives during the COVID-19 pandemic (Karlsson, 2021), highlighting how voluntary organizations played a pivotal role in both supporting and disseminating information to citizens, emphasizing the importance for surrounding social systems establishing collaborative practices to ensure there is a readiness to utilize their capacities in times of crisis. As noted by Hjorth et al. (2021), to optimize the contribution of non-profit organizations in future crises, it is essential for the state to define its expectations to ensure that they are utilized in the best possible way, while at the same time allowing them to operate in accordance with their role in society.

However, the current analysis indicates that public sector actors did not prioritize cooperation with associations and that associations had little agency and influence in advocacy activities such as user councils. Indeed, the findings highlight how public sector actors sometimes tended to avoid user councils, suggesting that collaboration prior to the pandemic was often limited to tokenistic gestures, where representatives of the service user movement were not acknowledged as valued partners in collaborative development processes (Beresford, 2020). Accordingly, in line with the international literature on user involvement during, and in the wake of the pandemic (Jones et al., 2020; Moreno et al., 2020), these findings underscore the urgent need for the surrounding welfare actors to make efforts to increase opportunities for collaboration and influence. This will help to endorse the influence of mental health service user organizations in the development of services and systems, in order to promote a more democratic and thus ethically strengthened provision of care and support.

Furthermore, the necessity for a consistent supply of resources to user organizations became evident. As highlighted in international research on the impact of the COVID-19 pandemic on not-for-profit organizations (Kim & Mason, 2020), the current findings emphasize the importance of having the opportunity to develop operating reserves in the form of funds set aside to provide a cushion against unexpected events that require additional resources in terms of staff, time and administration. A stronger commitment at government level would be needed to make associations less dependent on insecure annual subsidies from regions and municipalities. This would give them the opportunity to build sustainable infrastructures to maintain their member-supporting activities at times when public welfare actors are under such pressure that they are unable to fulfil their core tasks.

In conclusion, and in resonance with previous literature on disability advocacy organizations and digital communication (Gelfgren et al., 2022), the current findings underscore the importance of the welfare state in ensuring that all citizens have access to computers, the Internet, videoconferencing programmes that do not compromise confidentiality, and technological support to counter marginalization and the digital divide. In line with research that studied the need for technological development to ensure access to mental health care and support during the pandemic (Figueroa & Aguilera, 2020), the present analysis demonstrates the importance of clear government policies requiring the addressing of usability issues and the design of technology to meet the needs of people of various ages, technical abilities, languages, and levels of literacy. Furthermore, training for individuals with limited technological proficiency, through targeted outreach initiatives, is also recommended as a key strategy to mitigate the exacerbated marginalization of diverse communities (Figueroa & Aguilera, 2020).

### Limitations and suggestions for further research

In the collection of data, the heterogeneity of the sample composition of the study’s various interviews requires some comment. Our intention was to conduct separate interviews with board members and members separately throughout. Not least as there may be a power imbalance, limiting members’ willingness to criticize the board’s actions during the pandemic. However, in one instance, all participants were clear that they wanted to be interviewed together. While several members were forthcoming with their critical views, we cannot ascertain whether the discussions would have been even more candid in separate sessions. In regard to group size, we decided at an early stage to be flexible and prioritize access to as large a total number of participants as possible. Those unable to attend a scheduled focus group interview were offered the option to be interviewed individually. In a number of instances, only two or three participants took part, which may make it questionable to call them focus group interviews and not just group interviews. However, the high degree of interactivity observed in these conversations, we have chosen to retain the focus group concept. Given the varying numbers of participants on each occasion, the researchers took particular care to ensure that everyone had an equal opportunity to be heard. The focus group interviews with eight participants were permitted to last longer, with interruptions for breaks.

For future studies, we suggest longitudinal studies to track the long-term impact of the pandemic on these non-profit user organizations and their members. Moreover, as the theoretical analysis conducted in the current study was quite cursory, more in depth theoretical examinations may be suggested. As the findings highlighted power imbalances in terms of interaction with external organizations and risks of increased marginalization of vulnerable groups, it would be valuable to consider more critical perspectives on levels of participation (Arnstein, 1969) and co-optation (Selznick, 1980). Intersectional analyses may also be fruitful (Hankivsky et al., 2014), exploring the different difficulties and conditions that members of mental health service organizations face to be involved.

## Data Availability

The datasets generated during and/or analysed during the current study are not publicly available due to Swedish Publicity and Confidentiality Act but are available from the corresponding author on reasonable request. Ethical approval for the study was obtained from the Swedish Ethics Review Board (2021-05667-01).
